# Role of Human Leukocyte Antigen Class II in Antibody-Mediated Skin Disorders

**DOI:** 10.3390/medicina59111950

**Published:** 2023-11-04

**Authors:** Alvise Sernicola, Roberto Mazzetto, Jacopo Tartaglia, Christian Ciolfi, Paola Miceli, Mauro Alaibac

**Affiliations:** Dermatology Unit, Department of Medicine (DIMED), University of Padua, 35121 Padova, Italy; robertomazzetto93@gmail.com (R.M.); jacopo.tartaglia@studenti.unipd.it (J.T.); chriciolfi96@gmail.com (C.C.); paolamiceli9@gmail.com (P.M.); mauro.alaibac@unipd.it (M.A.)

**Keywords:** human leukocyte antigen, autoimmunity, autoantibody, autoimmune blistering disorder, pemphigus, pemphigoid, epidermolysis bullosa acquisita, dermatitis herpetiformis, linear IgA bullous disease

## Abstract

HLA class II molecules are key factors determining susceptibility to autoimmune disorders, and their role in immune-mediated skin conditions such as psoriasis has been extensively investigated. However, there is currently little understanding of their role in antibody-mediated skin diseases such as autoimmune blistering disorders. We researched the available literature using PubMed to narratively review the current knowledge on HLA associations in antibody-mediated blistering skin pathologies. Our results summarized the risk alleles that are identified in the literature, together with certain known protective alleles: in the pemphigus group, alleles HLA-DQB1*0503 and HLA-DRB1*0402 are most commonly associated with disease; in the pemphigoid group, the most studied allele is HLA-DQB1*0301; in epidermolysis bullosa acquisita, few genetic studies are available; in dermatitis herpetiformis, the association with haplotypes HLA-DQ2 and HLA-DQ8 is strongly established; finally, in linear IgA bullous disease, specific HLA alleles may be responsible for pediatric presentations. Our current pathogenic understanding of this group of disorders assigns a key role to predisposing HLA class II alleles that are able to bind disease autoantigens and therefore stimulate antigen-specific autoreactive T cells. The latter engage B lymphocytes that will produce pathogenic autoantibodies. The distribution of HLA alleles and their disease associations are variable across demographics, and an in-depth pathogenetic understanding is needed to support associations between HLA alleles and disease phenotypes. Additionally, in a personalized medicine approach, the identification of HLA alleles associated with the risk of disease may become clinically relevant in identifying susceptible subjects that should avoid exposure to known triggers, such as medication, when possible.

## 1. Introduction

Human leukocyte antigen (HLA) molecules are transmembrane glycoproteins expressed on nearly all nucleated cells in the body. The HLA region is located on the short arm of chromosome 6 (6p21), commonly referred to as the major histocompatibility complex (MHC). There are two major classes of HLA antigens: HLA class I (loci HLA-A, HLA-B, and HLA-C) and HLA class II (loci HLA-DR, HLA-DQ, HLA-DP, HLA-DM, and HLA-DO); these HLA molecules exhibit a high degree of polymorphism, with numerous alleles at each specific locus. The primary function of these glycoproteins is to regulate the immune response; in particular, class I and class II MHC molecules present peptides to CD8 cytotoxic T cells and to CD4 T cells, respectively. The HLA system has also gained prominent importance in the field of human tissue transplantation, where molecular HLA typing is routinely performed to allow for adequate matching between donor and recipient and to prevent rejection [1]. Paradoxically, susceptibility to autoimmune diseases has been consistently associated with MHC genotype, mostly MHC class II alleles. Several immune-mediated skin diseases have shown clear associations with specific HLA class II haplotypes, although the underlying mechanisms explaining how such HLA polymorphisms may confer susceptibility to certain diseases are still largely unknown.

## 2. Materials and Methods

The aim of our work is to review the current knowledge around HLA class II in antibody-mediated blistering cutaneous diseases, notably pemphigus group, pemphigoid group, dermatitis herpetiformis (DH), epidermolysis bullosa acquisita (EBA), and linear IgA bullous disease (LABD). To achieve this objective, PubMed was searched from inception to August 2023 using the terms “HLA” and “pemphigus” or “pemphigoid” or “dermatitis herpetiformis” or “epidermolysis” or “linear IgA disease”. Papers written in the English language were screened by the authors to retrieve results relevant to the topic of this review; moreover, the reference list of included papers was analyzed to identify additional results.

## 3. Pemphigus Group

HLA types play a crucial role in the development of pemphigus by facilitating the presentation of self-antigens to Th cells, activating humoral immunity, and generating self-antibodies. These antibodies disrupt intercellular adhesion, leading to the formation of erosions and blisters [2]. The two major categories of pemphigus are pemphigus vulgaris (PV) and pemphigus foliaceus (PF), accounting for 70% and 15–20% of all pemphigus cases, respectively. Less common variations of pemphigus are pemphigus herpetiformis (PH), paraneoplastic pemphigus (PNP), and IgA pemphigus (AP) [3]. The frequency of pemphigus diseases fluctuates across demographics, and this geographical distribution often aligns with specific HLA types within populations (Table 1).

### 3.1. HLA Associated with Pemphigus Vulgaris

PV involves autoantibodies targeting desmoglein 3 (Dsg3) and, occasionally, desmoglein 1 (Dsg1) [4]. The distribution of Dsg3 in the lower epidermal layers and epithelium makes mucous membranes the primary site of impact, while the presence of Dsg1 autoantibodies determines skin involvement [4]. There is an interesting variation in the types of HLA that are associated with PV. While some HLA types display population specificity, others link to PV across different ethnicities [5]. PV is commonly linked to alleles found on the HLA-A10, B38, DR4, DQw3.2 haplotype, prevalent among Jewish individuals [6,7,8]. HLA-DR4 polymorphism is governed by nucleotide sequence variability in the third hypervariable region of the DRB1 gene, specifically the Dw10 subtype on the A10, B38, DR4 haplotype that predisposes to pemphigus [9]. On the contrary, individuals lacking this DR4 haplotype generally carry HLA-DRw6 [2]. The two most common PV-associated alleles are HLA-DQB1*0503 and HAL-DRB1*0402, both of which were found to be associated with the disease in different populations [10]. In the Italian population, two PV susceptibility haplotypes were found: HLA-DRB1*0402, DQAl*0301, DQB1*0302 and HLA-DRB1*1401, DQA1*0104, DQB1*0503 [11]. The former was particularly widespread among Sardinian patients, integrated into an extended haplotype HLA-A2, Cw4, B35, S31, DR4, DQ8. Genetic resilience to PV seemed to be linked to the HLA-DR3, DQ2 haplotype within the Sardinian population [11]. Associations between PV and HLA class I alleles are observed in diverse populations: HLA-A3, HLA-A26, and HLA-B60 in Han Chinese; HLA-B38, HLA-C12, HLA-B57, and HLA-C15 in Brazilians; HLA-A10 and HLA-B15 in Japanese; HLA-B35 and HLA-B44 in Turks; HLA-B38 in Jewish and Spanish individuals; and HLA-B*4402, HLA-C*0401, and HLA-C*1502 in Iranians [12,13,14,15].

Population studies uncovered further links between specific class II alleles and PV within distinct ethnic groups. HLA-DRB1*0402 is associated with PV in over 90% of Ashkenazi Jews, while HLA-DQB1*0503 is associated with non-Jewish populations. HLA-DRB1*1404 emerges as an important risk factor within Indo-Asian populations. Furthermore, investigations highlight an association between PV and non-classic HLA class Ib alleles (HLA-E, HLA-F, and HLA-G). HLA g polymorphisms significantly associate with Jewish PV patients, while those of HLA-E—already implicated in multiple autoimmune conditions—are shared between Caucasian and Ashkenazi Jewish, potentially disrupting immune tolerance in these patients [12,13,14,15,16,17]. Apart from demographic variations, it is notable that PV affects females with a slight predominance, showing a female-to-male ratio of 1.5:1 [18]. This differential prevalence highlights potential sex-related genetic factors that may contribute to the development of PV. A recent analysis suggested a link between severe PV outcomes and specific HLA alleles-DRB1*0402 and DQB1*0302, which are predominant among female patients [5]. Familial occurrences of PV have also been documented, albeit infrequently [19]. These cases are rare and typically involve first-degree relatives in parent–child and/or sibling–sibling scenarios [20,21,22,23].

### 3.2. HLA Associated with Pemphigus Foliaceus

In PF, autoantibodies target Dsg1, while those targeting Dsg3 remain undetected. Consequently, PF primarily affects the skin, with mucosal surfaces having no involvement [24]. Endemic forms of PF have been identified in regions like Tunisia, Brazil, Peru, and Colombia, showcasing variations in incidence rates and sexual predisposition: Colombian PF displays a notable male prevalence [5,25]. Although PF is rare globally, it occurs with a high frequency in specific parts of South America: this confined variant, known as endemic pemphigus foliaceus or “fogo selvagem” in Brazil, shares clinical, immunological, and histological similarities with the sporadic form [26]. The existence of endemic subtypes underscores the influence of environmental factors in shaping their pathogenesis, as well as the significance of specific HLA imbalances within particular geographic regions. The significance of MHC class II alleles in determining susceptibility to PF was first recognized in 1989 following the demonstration of associations with HLA-DR and -DQ antigens. Haplotypes DR1–DQ1 and DR4–DQ3 were significantly increased in patients, while DR7–DQ2 and DR3–DQ2 were significantly decreased [27]. Subsequent analysis of HLA-DRB1 alleles and genotypes confirmed these associations and unveiled several risk alleles (e.g., DRB1*0101, *0102, *0103, *0404, *0406, *0410, *1406, and *1601) as well as protective alleles (e.g., *0301, *0701, *0801, *1101, *1104, and *1402) [28]. Notably, HLA-DQ allele DQw2 appears to be associated with factors preventing the development of disease in individuals living in endemic areas [29]. An analysis of 31 PF patients and 84 healthy controls confirmed the common DRB1*04 and DRB1*14 genetic background in French Caucasian PF patients. This study also identified susceptibility MHC class II alleles, including DRB1*0102, DRB1*0402, DRB1*0406, and DRB1*1404 [30]. In Italian patients, a shared susceptibility between PV and PF has been identified and is thought to reside in DRB1*1401 and DQB1*0503 susceptibility HLA alleles [31].

### 3.3. HLA Associated with Paraneoplastic Pemphigus

PNP is a type of pemphigus disease often associated with both malignant and benign neoplasms, frequently of hematologic, and lymphoid origin [5]. In PNP, autoantibodies typically target Dsg3 as well as various proteins within the plakin family, such as periplakin, envoplakin, plectin, desmoplakin 1 and 2, BP230, and the protease inhibitor alpha-2-macroglobulin-like-1 [32]. The genetic predisposition to PNP may exhibit variations among patients of different ethnicities and geographical regions. In Chinese patients, the HLA-Cw*14 allele has been proposed as a predisposing factor to PNP [33].

### 3.4. HLA Associated with Pemphigus Herpetiformis

PH is categorized as a clinical variant of pemphigus, merging the clinical attributes of DH with the immunopathological characteristics of pemphigus [34,35]. A case study detailing the development of PH subsequent to D-penicillamine treatment in a 57-year-old patient has been described [36]: HLA antigen typing identified HLA B8, A1, A2, and Bw39 [36].

### 3.5. HLA Associated with IgA Pemphigus

AP is marked by the presence of IgA autoantibodies targeting both desmosomal and non-desmosomal keratinocyte cell surface components. The predominant types within IgA pemphigus are subcorneal pustular dermatosis (SPD) and intraepidermal neutrophilic IgA dermatosis (IEN). In SPD, the autoantigen Dsc1 has been identified, whereas the antigen in the case of IEN is variable [37]. Associations between HLA classes and this specific type of pemphigus have not yet been described.

## 4. Pemphigoid Group

Autoimmune blistering disorders belonging to the pemphigoid group are defined by the presence of pathogenic autoantibodies targeting agents of the epithelial basement membrane zone (BMZ)—dermal epidermal junction or epidermal stromal junction—of the skin and mucosae. When there is a clinical suspicion, a specific diagnosis is confirmed by histology and direct immunofluorescence studies and supported by the serum detection of pathogenic autoantibodies. Histology is significant for subepidermal blister formation associated with a dermal infiltrate of neutrophils and eosinophils. In current clinical practice, serologic studies based on indirect immunofluorescence and ELISA may be sufficient for diagnosis, especially when the clinical presentation is clearly suggestive. The HLA-DQβ1*0301 allele (HLA-DQ7) has been reported as a shared susceptibility gene for all subsets in the pemphigoid group (Table 2). To support the notion that a single HLA allele is responsible for binding different antigens across pemphigoid disease subsets, predictive computational models have been employed to identify potential binding sites for DQβ1*0301 on BP180, BP230 and on integrins alpha 6 and beta 4 [38].

### 4.1. HLA Associated with Bullous Pemphigoid

Bullous pemphigoid (BP) usually affects elderly subjects and is the most commonly observed autoimmune blistering disorder (AIBD), partly owing to the constant aging of the general population. Its management may be challenging owing to the fragility and frequent comorbidities of patients, which may limit therapeutic options and increase overall morbidity. The onset of disease is the result of an interaction between genetic predisposition and potential triggering factors, including certain drugs. The role of HLA in mediating predisposition to autoantibody-mediated skin blistering conditions is established in the literature, with HLA class II genes such as DR and DQ reporting high prevalence in subjects with BP [5]. HLA-DQB1*0301 constitutes the most strongly established disease association in Caucasians and other ethnicities—including in Brazilian and Iranian studies [39,40,41,42]—but not in studies on Japanese and Northern Chinese populations [43,44]. However, according to one study, this association was restricted to men [45]. Moreover, DQA1*0505 was associated with Brazilian, Japanese, and Han Chinese groups, as well as in a German study, together with DRB1*0701 [46]. A recent metanalysis highlighted that HLA-DQA1*0505 is associated with an increased odds of PB, while DQA1*0201 carries protection from this disease [47]. Drug-induced BP may be triggered following exposure to certain drug classes, principally inhibitors of dipeptidyl peptidase 4 (DPP-4i) for the treatment of diabetes mellitus and oncologic immunotherapy with inhibitors of the PD-1 immune checkpoint [48,49]. Japanese patients who presented BP during treatment with DPP-4i had the following risk alleles: DQB1*0301, DQA1*0505, DRB1*1101, and DRB1*1201. In the same study, two additional alleles were associated with protection: DQA1*0103 and DQB1*0601 [50].

### 4.2. HLA Associated with Mucous Membrane Pemphigoid

The definition of mucous membrane pemphigoid (MMP) encompasses a heterogeneous disease phenotype with two key features: a predilection for mucosal involvement and a scarring tendency, which explains its progressive course, leading occasionally to serious sequelae. This is particularly relevant for ocular cicatricial pemphigoid, where scarring affects the conjunctival surfaces, leading to severe vision impairment. Autoantibodies targeting different antigens of the BMZ are detected in MMP: BP180, laminin 332, and integrin beta4 subunit in ocular MMP. The HLA class II allele most studied in MMP is DQB1*0301, the association of which has been highlighted in Caucasian populations and in subsets of patients with ocular cicatricial pemphigoid [51,52,53]. Additional specific HLA class II alleles in MMP are DRB1*04 and DRB1*11 [54].

### 4.3. HLA Associated with Pemphigoid Gestationis

Pemphigoid gestationis (PG) is a rare though well-characterized dermatosis of pregnancy that usually develops in the later periods of gestation or postpartum. A cutaneous vescicobullous eruption is the result of IgG1 autoantibodies produced against BP180. The development of disease is related to HLA class II mismatch in the placenta, and specifically to maternal exposure to paternal tissue, being the placental genome of paternal origin [55]. PG has been related to maternal HLA-DR3 and DR4 haplotypes and with DRB1*0301 and DRB1*0401/040X polymorphisms [56].

## 5. Epidermolysis Bullosa Acquisita

EBA is a rare diagnosis, associated with a spectrum of clinical variants: a “classical” presentation consisting of an acquired, chronic, trauma-induced, subepidermal blistering disease; a BP-like presentation; and other variants with features reminiscent of linear IgA dermatosis or MMP [57,58]. The disease results from pathogenic antibodies against collagen VII in the dermal–epidermal junction. Due to the rarity of patients, human genetic studies on this condition are sparse and support the role of EBA-predisposing genetic factors in certain populations (Table 3). EBA may be more common in Asians [59,60,61], and a significant association with HLA-DRB1*13 was found in Koreans [62]: 6/12 (50%) EBA patients versus 22.3% of the controls were reported to have this allele. In the USA, the first large series of EBA patients comprised 18/29 (62%) Black patients of African descent, suggesting a predisposition in African Americans. An association was found within this group with HLA-DRB1*15 (HLA-DR2) [63], and a subsequent study reported associations with the alleles DRB1*1503 and DQB1*0602, which is in linkage disequilibrium with the former [64].

## 6. Dermatitis Herpetiformis

DH is a chronic, autoimmune, blistering skin disorder intrinsically connected to gluten sensitivity [65]. Currently, DH is acknowledged as the characteristic skin manifestation of celiac disease (CD) [66]. DH and CD share several characteristics, including gluten sensitivity, strong associations with specific human HLA haplotypes (Table 3), and the presence of circulating antibodies against tissue and epidermal transglutaminase [65]. Histologically, both disorders exhibit comparable features, including characteristic small-bowel villous atrophy. Although the majority of DH patients display varying degrees of villous atrophy, approximately 20% exhibit normal mucosal architecture accompanied by inflammatory alterations suggestive of latent CD [67,68]. Adherence to a gluten-free diet results in improvement in both the cutaneous eruption and the enteropathy [69]. The clinical manifestation of DH involves pruritic, symmetrical, and polymorphic lesions progressing from erythema to urticarial plaques, papules and vesicles, commonly involving extensor surfaces of the limbs [70,71,72]. The complex interplay between genetic susceptibility and environmental factors significantly contributes to the pathogenesis of DH, with a pivotal role attributed to the HLA system underlying its genetic foundation [73]. The genetic basis of DH is apparent from twin studies. A prospective study involving six sets of monozygotic twin pairs demonstrated a concordance rate of 0.91, exceeding expectations for a complex hereditary trait [74]. However, the significance of these findings is limited by the study’s small sample size, which does not allow for comprehensive statistical analysis. This hereditary component is further elucidated by a population-based analysis revealing nearly 15-fold higher CD and DH incidence in first-degree relatives, underscoring the significant influence of genetic factors. In this study, it was found that up to 18% of individuals affected by DH had a first-degree relative who also exhibited either DH or gluten intolerance [75]. Research by Spurkland et al. illustrates the weight of HLA-DQ2 and HLA-DQ8 in the genetic landscape of DH [76]. In their study, 50 individuals with DH were compared to 289 healthy controls. The results indicated that 86% of the DH patients carried the HLA-DQ2 haplotype (combination of the DQA1*0501 and DQB1*02 alleles), while 12% had the HLA-DQ8 haplotype (combination of the DQA1*03 and DQB1*0302 alleles). The presence of either or both haplotypes correlated with a sensitivity close to 100% for both CD and DH. Conversely, individuals lacking these haplotypes exhibited minimal likelihood of developing CD and DH [76]. These findings strongly reinforce the genetic connection between these two disorders. The experimental validation of HLA associations with DH is provided by murine models. Among 90 NOD (Non-Obese Diabetic) DQ8+ mice subjected to gluten sensitization, 15 displayed blistering pathology resembling DH [77]. This was characterized by neutrophil infiltration into the dermis, IgA deposition at the dermal–epidermal junction, and the complete resolution of blistering upon implementing a gluten-free diet, with or without dapsone treatment. The incorporation of DQ8 heightened sensitivity to gliadin, while the NOD background augmented autoimmune susceptibility. Early genetic investigations conducted in the 1970s and 1980s, which suggested links between HLA-A1, HLA-B8, and HLA-DR3 haplotypes with both DH and CD, exhibit varying levels of reproducibility [78,79,80,81]. These associations seem relatively less pronounced when compared to the robust connections observed with HLA-DQ2 and HLA-DQ8 haplotypes. DH displays varying prevalence across different ethnic groups, being most common in patients of Northern European descent, rare among Asians, and even rarer among African Americans [65]. These demographic variations are linked to the relationship between DH and specific HLA alleles within diverse populations. DH patients of Asian descent, particularly in Japan, exhibit distinct characteristics if compared to Caucasian subjects, partly due to the absence of the HLA-DQ2/DQ8 haplotypes [82]. Exploring the Chinese population, a genetic study involving genome-wide variant analysis and HLA typing of DH was conducted by Sun et al. [83]. Notably, HLA-B*0801 and HLA-DRB1*0301 were identified as risk alleles for DH in this population, reaffirming the pivotal role of HLA in its development. Additionally, the study revealed a protective effect of HLA-DRB1*0301 against gastrointestinal symptoms. An assessment of the practical utility of these identified risk variants was carried out using a comprehensive analysis of all sequenced samples. It was observed that HLA-B*0801 was found in 36.1% of patients diagnosed with DH, in contrast to its presence in 1.5% of the healthy controls. Conversely, the presence of DRB1*0301 exhibited a sensitivity of 50% and a specificity of 93.3% in predicting the risk for DH. Incorporating both of these risk variants led to an enhancement of the predictive capability, albeit to a moderate extent [83]. While HLA-DQ2 and HLA-DQ8 are cornerstones of DH genetic architecture, emerging research has identified candidate genes like myosin IXB (MYO9B) as potential contributors [84,85,86]. MYO9B’s mutations are implicated in intestinal barrier integrity and permeability, thus affecting gluten penetration and immune responses [85]. Although the evidence of association with DH is less robust, these findings shed light on the genetic landscape of DH beyond HLA. The autoimmune nature of DH and BP is underscored by cases where both conditions co-occur in patients with specific HLA alleles associated with each disorder [39]. Notably, genetic convergence of HLA alleles—such as HLA-DQB1*0301 (predisposing to BP), HLA-DQA1*0505/0501 (predisposing to CD), and HLA-DQB1*0201 (predisposing to both CD and DH)—highlights distinct genetic predispositions within the same patient. This emphasizes the genetic basis behind their simultaneous occurrence, highlighting the intricate role of genetic factors in shaping autoimmune disorders.

## 7. Linear IgA Bullous Disease and Chronic Bullous Disease of Childhood

Linear IgA bullous disease (LABD) is a rare autoimmune blistering dermatosis with linear IgA deposits along the basement membrane zone. It occurs after puberty, with most patients presenting after the age of 60. Clinically, it manifests with pruritic papules, vesicles, and bullae, usually distributed on extensor surfaces; mucosal involvement is common, with erosions or ulcers and, sometimes, mucosal scarring [87]. In the pediatric population, LABD is known as a chronic bullous disease of childhood (CBDC), presenting predominantly in children younger than 5 years of age. Clinically, it is characterized by tense bullae on an inflammatory base, mainly localized to the lower abdomen and the perineal and perioral areas; lesions are often grouped in a “cluster of jewels” appearance, with new lesions appearing at the periphery of older lesions. Mucosal involvement is less frequent and usually less severe than in adults [88]. Most cases of LABD and CBDC are idiopathic; however, several cases of drug-induced LABD have also been reported in the literature, with vancomycin being the most frequently associated medication. Most patients with LABD and CBDC have IgA1 antibodies targeting 97-kDa (LABD-97) and 120-kDa (LAD-1) antigens, which are both fragments of BP180; however, in some cases, antibodies targeting other antigens (like the NC16A epitope on BP180, type VII collagen, and laminin-332) can be identified, probably due to the epitope spreading phenomenon [87,88]. Genetic factors may also contribute to the development of LABD and CBDC. Associations of LABD with HLA-B8 have been reported in several studies [89,90,91], whereas others have found no significant association [92,93]. Wojnarowska et al. reported an increased frequency of HLA-B8 in CBDC and LABD in the UK, with a significantly higher incidence in CBDC (76%) compared to LABD (28%) [94]. An even higher percentage of HLA-B8 positivity (80%) was found in Tunisian children [95]. Collier et al. also found an increased frequency of HLA-B8, DR3, and DQ2 in CBDC but not in adult LABD, suggesting that a carrier state of these alleles could explain the earlier presentation of skin lesions in children with CBDC. The authors also found a significant increased frequency of HLA-Cw7 in both adults and children [89] (Table 3).

## 8. Discussion and Conclusions

The prominent involvement of HLA genes in the initiation of AIBDs is supported by the key role of autoreactive Th2 cells in the pathogenesis of this group of disorders, that is ultimately characterized by specific autoantibody production. Autoreactive T cells are considered crucial for disease initiation but are also detected in healthy individuals; therefore, blistering diseases may occur following an imbalance between Th2 responses against cutaneous antigens, which are associated with disease, and Th1 regulatory responses, which maintain immune tolerance and are dysfunctional in disease [96]. According to the current understanding, the characteristic disease antigens are bound by predisposing HLA class II alleles and stimulate antigen-specific autoreactive T lymphocytes. The latter then engage, through CD40-CD40L interaction, the B lymphocytes that will secrete specific antibodies targeting these autoantigens. In the case of BP, similar epitopes of BP180—which is the major autoantigen for BP—have been shown to be recognized by both autoreactive Th cells and IgG4 autoantibodies [97].

Genetic associations of HLA genes with AIBDs have been reviewed to highlight alleles that are related to increased susceptibility or to protection from the development of these disorders (Table 4). These associations are variable across demographics, reflecting the differential distribution of HLA alleles between populations and the geographical differences in the frequency of antibody-mediated blistering disorders.

Subjects that carry susceptibility genes to autoimmunity are particularly exposed to developing AIBDs when exposed to triggering factors, such as certain medications. Therefore, the identification of HLA alleles associated with the risk of disease may become clinically relevant in a personalized medicine approach: specific genetic testing may allow for a better patient selection and avoid the exposure of predisposed subjects to trigger drugs whenever possible.

Additionally, variability in HLA class II alleles is able to influence the quantity and specificity of autoantibodies that are produced in these disorders. An in-depth understanding of their pathogenesis is needed to support associations between HLA alleles and severe disease phenotypes. Finally, the onset of autoimmune comorbidities in subjects with AIBD and the possibility of simultaneous disorders mediated by two different antibodies may be dependent on the co-occurrence of multiple HLA risk alleles. This may explain an epitope spreading phenomenon, through which an antigen can be presented to the immune system by two different HLA class II molecules and trigger the secretion of two different autoantibodies [38].

## Figures and Tables

**Table 1:** Main HLA associations in the pemphigus group of disorders, grouped according to geographic- and population-specific differences.

Disease	HLA-DR	HLA-DQ	Notes
Pemphigus vulgaris	HLA-DR4	HLA-DQB1 *0503	
	HLA-DRw6		
	HLA-DRB1 *0402		
	HLA-DR4	HLA-DQw3.2	Jewish
	HLA-DRB1		Ashkenazi
	HLA-DRB1 *0402 *1401	HLA-DQB1 *0302 *0503 HLA DQA1 *0104 *0301	Italian
	HLA-DRB1 HLA-DR4 *0402	HLA-DQA1 *0301	Sardinian
	HLA-DRB1 *1404		Indo-Asian
Pemphigus foliaceus	HLA-DR1	HLA-DQ1	
	HLA-DR4	HLA-DQ3	
	HLA-DRB1 **		
	HLA-DRB1 *04 (*0402, *0406) *14 (*1404) *0102		French
	HLA-DRB1 *1401	HLA-DQB1 *0503	Italian

Abbreviations: HLA, human leukocyte antigen—DR, DQ isotypes. ** HLA-DRB1*0101, *0102, *0103, *0404 *0406, *0410, *1416, *1601.

**Table 2:** Main HLA associations in the pemphigoid group of disorders grouped according to geographic- and population-specific differences.

Disease	HLA-DR	HLA-DQ	Notes
Pemphigoid group		HLA-DQ7: HLA-DQβ1 *0301	
	HLA-DRB1 *0701	HLA-DQA1 *0505	Brazilian, Japanese, Han Chinese, German
	HLA-DRB1 *1201	HLA DQ7: HLA-DQβ1 *0301 HLA-DQA1 *0505	Japanese patients treated with DPP-4i
Mucous membrane pemphigoid	HLA-DRB1 *04 *11	HLA-DQ7: HLA-DQβ1 *0301	
	HLA-DRB1 *0301		
Pemphigoid gestationis	HLA-DR3 HLA-DR4 HLA-DRB1 *0401/040X		Maternal HLAs

Abbreviations: HLA, human leukocyte antigens—DR, DQ isotypes; DPP-4i, dipeptidyl peptidase 4 inhibitors.

**Table 3:** Main HLA associations in epidermolysis bullosa acquisita, dermatitis herpetiformis, and chronic bullous disease of childhood, grouped according to geographic- and population-specific differences.

Disease	HLA-DR	HLA-DQ	Notes
Epidermolysis bullosa acquisita	HLA-DRB1 *13		Koreans
	HLA-DR2, HLA-DRB1*15 HLA-DRB1 *1503	HLA DQB1 *0602	African Americans
Dermatitis herpetiformis	HLA-DR3	HLA-DQ2: HLA-DQA1 *0501, HLA-DQB1*02	
	HLA-DR3	HLA-DQ8: HLA-DQA*03, HLA-DQB1*0302	
	HLA-DRB1 *0301		Chinese
Chronic bullous disease of childhood	HLA-DR3	HLA-DQ2	

Abbreviations: HLA, human leukocyte antigen—DR, DQ isotypes.

**Table 4:** HLA alleles with a recognized protective role against the development of autoimmune blistering disorders.

Disease	HLA-DR	HLA-DQ	Notes
Pemphigus group			
Pemphigus vulgaris	HLA-DR3	HLA-DQ2	Sardinian
Pemphigus foliaceus	HLA-DR3 HLA-DR7 HLA-DRB1 **	HLA-DQ2 HLA-DQw2	
Pemphigoid group			
Bullous pemphigoid		HLA-DQA1 *0201 *0103 HLA-DQB1 *0601	
Dermatitis herpetiformis			
	HLA-DRB1 *0301		Gastrointestinal symptoms

Abbreviations: HLA, human leukocyte antigen—DR, DQ isotypes. ** HLA-DRB1*0301, *0701, *0801, *1101 *1104, *1402.

## Data Availability

No new data were created or analyzed in this study. Data sharing is not applicable to this article.
